# Slit3 by PTH-Induced Osteoblast Secretion Repels Sensory Innervation in Spine Porous Endplates to Relieve Low Back Pain

**DOI:** 10.21203/rs.3.rs-4823095/v1

**Published:** 2024-08-31

**Authors:** Janet Crane, Weixin zhang, Arryn Otte, Sisir Barik, Mei Wan, Xu Cao

**Affiliations:** Johns Hopkins Univeristy School of Medicine; The Johns Hopkins University School of Medicine; Johns Hopkins University; Johns Hopkins University; Johns Hopkins University; Johns Hopkins University School of Medicine

## Abstract

During aging, the spine undergoes degenerative changes, particularly with vertebral endplate bone expansion and sclerosis, that is associated with nonspecific low back pain (LBP). We reported that parathyroid hormone (PTH) treatment could reduce vertebral endplate sclerosis and improve pain behaviors in aging, SM/J and young lumbar spine instability (LSI) mice. Aberrant innervation noted in the vertebral body and endplate during spinal degeneration was reduced with PTH treatment in aging and LSI mice as quantified by PGP9.5^+^ and CGRP^+^ nerve fibers, as well as CGRP expression in dorsal root ganglia (DRG). The neuronal repulsion factor Slit3 significantly increased in response to PTH treatment mediated by transcriptional factor FoxA2. PTH type1 receptor (PPR) and Slit3 deletion in osteoblasts prevented PTH-reduction of endplate porosity and improvement in behavior tests, whereas PPR deletion in chondrocytes continued to respond to PTH. Altogether, PTH stimulates Slit3 to repel sensory nerve innervation and provides symptomatic relief of LBP associated with spinal degeneration.

## Introduction

Low back pain (LBP) is one of the most common skeletal pain diseases especially in the aging population. Chronic low back pain profoundly affects the quality of life and daily physical activity and is a crucial risk factor for future health decline^[1–3]^. Most LBP is nonspecific with no apparent pathoanatomical cause^[4–7]^, which can be attributed to a diverse range of reasons, including biological, psychological, and social factors^[1, 8]^. Studies indicate that the prevalence of low back pain peaks at 28–42% among individuals between the ages of 40 and 69. In the USA, the annual cost associated with LBP management surpasses 100 billion dollars^[9]^. A notable pathological feature of low back pain is the nociceptive innervation of the spine, impacting structures such as muscles, ligaments, and especially vertebral endplate. The primary therapeutic approaches encompass behavioral management, pharmacological treatments like non-steroidal anti-inflammatory drugs (NSAIDs) or muscle relaxants, and surgical interventions, all aimed at maintaining function^[10]^.

In recent years, we have demonstrated that during aging, endplates undergo calcification while osteoclasts generate porosity stimulating aberrant sensory innervation. Specifically, osteoclasts in the porous endplates secrete factors that induce sensory innervation to cause LBP^[11, 12]^. The cartilaginous endplate is composed of a thin layer of hyaline cartilage positioned between the vertebral endplate, the coronal surface of each vertebra, and the nucleus pulposus, which is the inner core of the vertebral disc that acts as the shock absorber for each spinal unit^[13, 14]^. Endplates are cartilaginous with no blood vessels and nerve fibers, and the environment in the porous endplates is very acidic. We have uncovered that the attractive neuronal guidance factor, Netrin-1, secreted by osteoclast lineage could induces sensory innervation in porous endplates and mediates low back pain^[11, 15]^. Importantly, increased senescence osteoclasts and macrophages in the porous endplates secrete Netrin-1 and elimination of senescent cells with a senolytic drug could significantly decrease sensory innervation and to reduce LBP^[16, 17].^

Aging of the musculoskeletal system results in chronic skeletal pain, especially in conditions of such as osteoarthritis (OA), and spinal degeneration ^[18–20]^. Pain is a process by which noxious stimuli are converted into electrical signals by different receptors or channels in specialized sensory neurons called nociceptors^[21, 22]^. Once the nociceptor is sufficiently activated, the electrical signal is transmitted along the nerve fibers towards the spinal cord and, eventually, the brain^[23]^. As the pain signal travels, its strength and character can be modulated by various factors in different regions^[24, 25]^. In recent years, the emerging concept of skeletal interoception has shed light on the regulation of nociceptive innervation triggered by prostaglandin E2 (PGE2) in osteoarthritis and spinal hypersensitivity^[26]^. Beyond the sensitization of sensory nerve fibers by inflammatory stimuli, active osteoclasts can further promote sensory innervation in the subchondral bone or spinal endplate porous regions via Netrin-1 and DCC, amplifying pain signaling^[11, 27]^. Therapies that block the PGE2 pathway, whether through cyclooxygenase-2 (COX-2) inhibitors or sensory nerve blockers, can notably alleviate pain^[12]^.

Parathyroid hormone (PTH) is produced and secreted by the parathyroid glands. It plays an essential role in the regulation of calcium and phosphate metabolism, as well as in bone metabolism^[28]^. Intermittent administration of PTH primarily stimulates bone formation, whereas continuous elevation of PTH significantly promotes bone resorption^[29]^. Our research has shown that PTH treatment impacts not only bone structural remodeling but also alleviates osteoarthritis pain and spinal hypersensitivity in animal models by promoting osteoblastic bone formation in the porous endplates and reduces PGE2 levels^[30–33]^. However, the mechanism by which PTH treatment reduces sensory denervation in the porous endplates remains unclear. During development, the distribution of nerve fibers is orchestrated by various guiding factors. These factors ensure that nerve fibers, also known as axons, navigate accurately to their designated targets, thereby establishing functional neural circuits. The primary guiding factors include Netrins, Slits, Semaphorins, Ephrins, Neurotrophins, and others, which can be secreted by diverse sources, such as neurons, endothelial cells, immune cells, osteoblasts, and osteoclasts^[15, 34, 35]^. While the mechanisms by which guiding factors regulate sensory innervation or denervation and their subsequent influence on pain in skeletal diseases, such as low back pain, remain elusive. In the current research, we found that PTH stimulated Slit3 secreted by osteoblasts to function as a repulsive factor to sensory innervation, reducing LBP.

## Results

### Degenerated endplate structure and pain behavior are improved by PTH treatment

**Figure 1:**
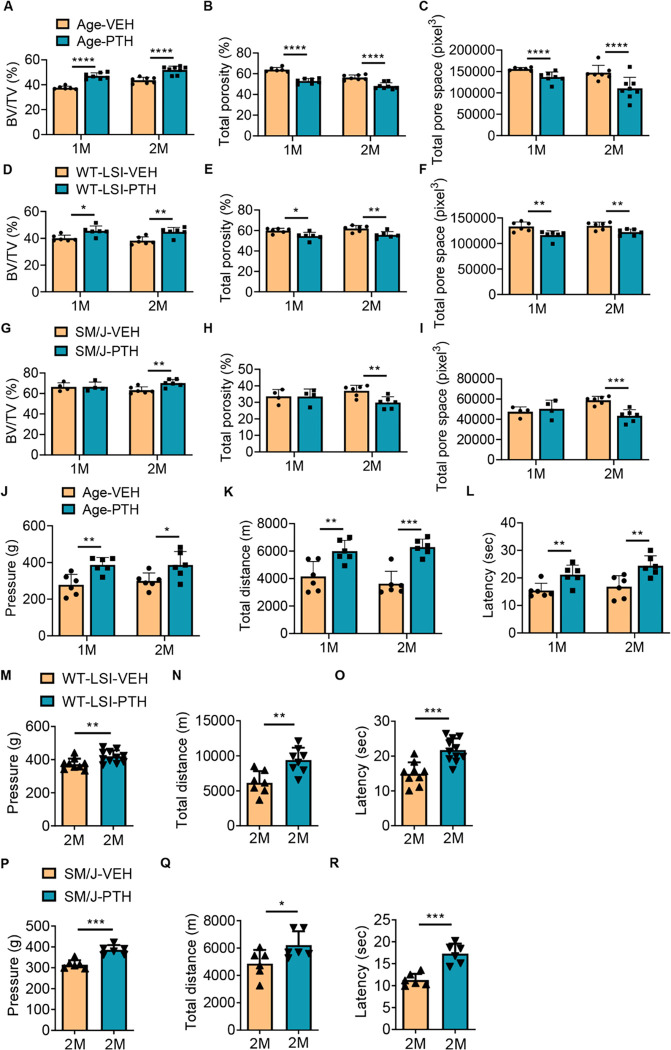
Micro-CT and behavior analysis of three spinal degeneration mouse models. **(A-C)** Vertebral endplate bone structure analyses by micro-computed tomography: Bone volume/Tissue volume (A), Total porosity percentage (B) and Total pore space (C) of fifth lumbar (L5) endplate of aging mice treated with parathyroid hormone (PTH) (40 μg/Kg/day) for one or two months relative to vehicle-treated (Veh) group. (n ≥ 6, t-test). **(D-F)** Bone volume/Tissue volume (D), Total porosity percentage (E) and Total pore space (F) of L5 endplate of WT young mice two months after lumbar spine instability (LSI) surgery and treated with PTH (40 μg/Kg/day) for one or two months relative to Veh-group. (n ≥ 6, t-test). **(G-I)** Bone volume/Tissue volume (G), Total porosity percentage (H) and Total pore space (I) of L5 endplate of SM/J mice treated with PTH (40 μg/Kg/day) for one or two months relative to Veh-group. (n ≥ 4, t-test). **(J-L)** Behavior evaluations included pressure tolerance in the lumbar spine region as determined by force threshold (J), total distance covered during spontaneous activity in two days (K), and latency of hind paw withdrawal post-thermal stimulation (L) in aging mice treated with PTH or a Veh for one or two months. (n ≥ 6, t-test). **(M-O)** Pressure tolerance in the lumbar spine region (M), total distance covered during spontaneous activity in two days (N), and latency of hind paw withdrawal post-thermal stimulation (O) in WT young mice two months after LSI surgery and treated with PTH or Veh for two months. (n ≥ 8, t-test). **(P-R)** Pressure tolerance in the lumbar spine region (P), total distance covered during spontaneous activity in two days (Q), and latency of hind paw withdrawal post-thermal stimulation (R) in SM/J mice treated with PTH or Veh for two months. (n ≥ 6, t-test). *P 0.05, **P 0.01, ***P 0.005, ****P 0.001.

To assess the efficacy of PTH treatment regarding low back pain, we utilized 3 spinal degeneration models: 1) aging of C57BL/6J (WT) strain mice, 2) young WT mice two months after lumbar spine instability (LSI) surgery to stimulate mechanical injury in the development of low back degeneration (Supplemental Fig. 1A), and 3) SM/J transgenic mice as a model of accelerated aging. Over the duration of two weeks, one or two months, mice were administered PTH (40 μg/Kg/day) or vehicle daily via intraperitoneal (IP) injection. Bone quality of the lumbar five (L5) spine endplate was evaluated using micro-CT scanning. Our findings revealed significant changes in the spine endplate morphology by 1 month for both the aging and LSI mouse models, whereas similar changes were not observed until after 2 months of PTH treatment in the SM/J model relative to vehicle controls. Specifically, there was a significant increase in bone volume and decreased total porosity and pore space of the L5 endplate in PTH group relative to vehicle group (Fig. 1, A–I; Supplemental Fig. 1, B-G). To ascertain whether PTH treatment alleviated chronic spinal pain in our experimental models, we subjected aging, WT LSI, and SM/J mice to a series of behavioral tests including hyperalgesia to applied pressure, spontaneous wheel running, and thermal tolerance. In aging mice, PTH treatment for durations of one and two months, but not two weeks, showed a notable improvement in hyperalgesia pressure tolerance of lumbar spine and in the total distance of spontaneous running in the active-wheel (Fig. 1, J–K, Supplemental Fig. 1H). All three PTH treatment durations significantly extended the latency of the hind paw withdraw to the thermo stimulation of Hargreaves test in aging mice relative to vehicle (Fig. 1L, Supplemental Fig. 1I). Behavior tests in WT LSI and SM/J mice after two months of PTH administration demonstrated significant improvement in pressure tolerance, total distance on spontaneous wheel running, and enhanced the tolerance to thermo stimulation relative to vehicle controls (Fig. 1, M–R). Collectively, these observations underscore that PTH treatment can mitigate chronic spinal pain and rescue the endplate structure across various mouse models of spinal degeneration.

### Nociceptive innervation decreased in PTH treated mice

**Figure 2:**
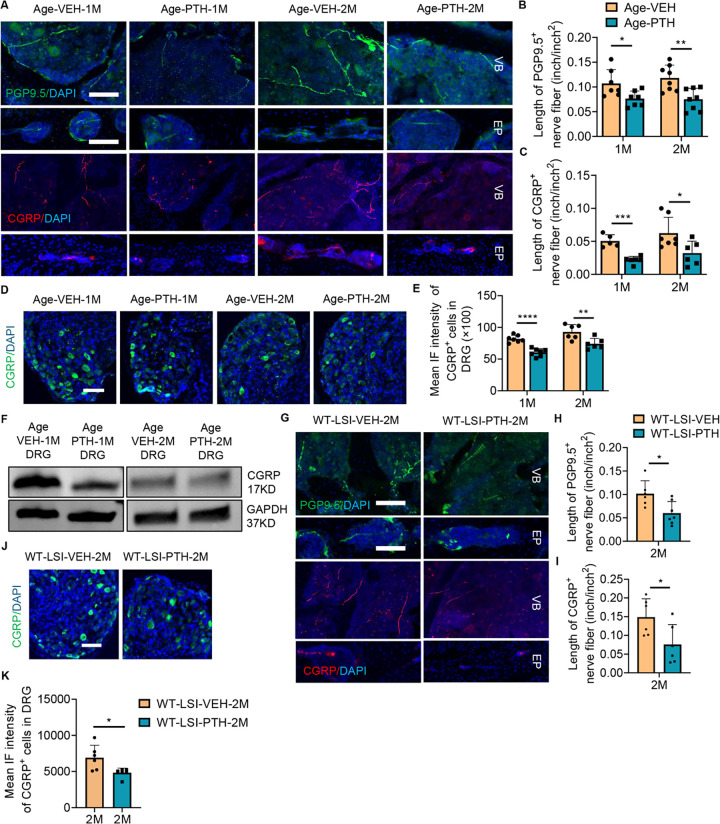
PTH Treatment Alters Innervation in the Degenerated Spine. **(A)** Representative images of PGP9.5-positive fibers (upper row, Green) and CGRP-positive fibers (lower row, Red) in the lumbar vertebral body and endplate of aging mice treated with parathyroid hormone (PTH) or vehicle (Veh) for one or two months. Scale bar: 100 μm. **(B-C)** Quantitative analysis of the length of PGP9.5-positive fibers (B) or CGRP-positive fibers (C) in the lumbar vertebral body of aging mice treated with PTH or Veh for one month or two months (n ≥ 5, t-test). **(D-E)** Representative images (D) and quantitative mean immunofluorescent (IF) intensity (E) of CGRP-positive neurons in the dorsal root ganglia (DRG) (L1-L2) of aging mice treated with PTH or Veh for one month or two months. Scale bar: 100μm. (n ≥ 6, t-test). **(F)** Protein levels of CGRP in DRG tissue (L1-L2) relative to the expression of GAPDH in aging mice treated with PTH or Veh for one or two months, respectively. (n = 5). **(G-I)** Representative images and quantitative analysis of the length of PGP9.5-positive (G, H, Green) and CGRP-positive (G, I, Red) fibers in the vertebral body and endplate of WT young mice two months after LSI surgery and treated with PTH or Veh for two months. Scale bar: 100μm. (n ≥ 6, t-test). **(J-K)** Representative images (J) and quantitative mean IF intensity (K) of CGRP-positive neurons in the DRG of WT young mice two months after LSI surgery and treated with PTH or Veh for two months. Scale bar: 100μm. (n ≥ 5, t-test). DAPI stains nuclei blue. EP: endplate, VB: vertebral body. *P 0.05, **P 0.01, ***P 0.005, ****P 0.001.

**Figure 3:**
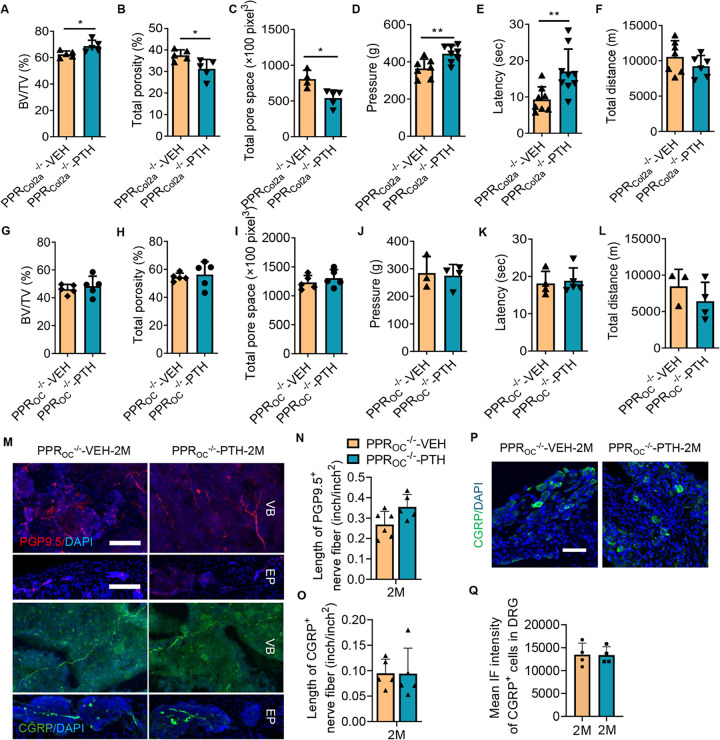
Osteoblast Stimulation by PTH Counteracts Spine Degeneration. **(A-C)** Vertebral endplate bone structure analyses by micro-computed tomography: BV/TV percentage (A), Total porosity percentage (B), and Total pore space (C) in mice with conditional knockout of the type 1 parathyroid hormone (PTH) type 1 receptor (PPR) in type II collagen (Col2a) expressing cells (PPR_Col2a_^ERT2−/−^) that underwent lumbar spine instability (LSI) surgery for two months and treated with PTH or vehicle (Veh) for an additional two months. (n = 5, t-test). **(D-F)** Behavior evaluation of PPR_Col2a_^ERT2−/−^ mice post-LSI surgery for two months, then treated with PTH or Veh for another two months included pressure tolerance assessed by force threshold (D), latency of paw withdrawal post-thermal stimulation (E), and total spontaneous activity distance traveled in two days (F). (n ≥ 7, t-test). **(G-I)** BV/TV percentage (G), Total porosity percentage (H), and Total pore space (I) in mice with conditional knockout of PPR in Osteocalcin (OC) expressing cells (PPR_OC_^−/−^) subjected to LSI surgery two months prior to daily treatment with PTH or Veh for two months. (n = 5, t-test). **(J-L)** Behavior evaluation of PPR_OC_^−/−^ mice post-LSI surgery for two months, then treated with PTH or Veh for another two months included pressure tolerance of the lumbar spine region assessed by force threshold (J), latency of paw withdrawal post-thermal stimulation (K), and total spontaneous activity distance traveled in two days (L). (n ≥ 3, t-test). **(M-O)**Representative images of PGP9.5 and CGRP-positive fibers (M), quantitative analysis of fiber length in the lumbar vertebral body of PPR_OC_^−/−^ mice post-LSI and post-PTH or Veh treatment (N, O). Scale bar: 100μm. (n ≥ 5, t-test). **(P-Q)** Representative images showing CGRP-positive neuron (P), quantitative analysis of mean intensity of immunofluorescence (Q) in the dorsal root ganglia of PPR_OC_^−/−^ mice post-LSI and post-treatment. Scale bar: 100μm. (n = 4, t-test). DAPI stains nuclei blue.

To uncover the mechanism by which PTH treatment alleviates pain, we examined peripheral sensory innervation. Notably, we found a significant reduction in peripheral sensory nerve innervation marked by PGP9.5 and CGRP-positive nerve fibers within the vertebral body and endplate in aging mice treated with PTH for one month, and two months, but not two weeks, relative to vehicle control (Fig. 2, A–C, Supplemental Fig. 2A, B). In-depth examination of CGRP expression in Lumbar 1 or Lumbar 2 (L1/L2) dorsal root ganglion (DRG) showed a pronounced decrease in neurons after one and two months of PTH administration in aging mice (Fig. 2, D–E). Decreased protein levels of CGRP and PGP9.5 in DRG was further confirmed by Western blot in aged mice treated with PTH for one month compared with vehicle controls (Fig. 3F, Supplemental Fig. 2C). Similar results were observed in young WT LSI mice treated with PTH relative to vehicle controls. Specifically, the relative length of PGP9.5-positive and CGRP-positive fiber in the vertebral body, and expression of CGRP in the L1/L2 DRG were significantly decreased in the WT LSI mice treated with PTH for two months relative to vehicle control (Fig. 2, G–K). PTH treatment also reduces the expression of Isolectin IB_4_ (IB4) and Tyrosine hydroxylase (TH) in spinal endplate (L5) relative to vehicle control in aged mice (Supplementary Fig. 2D), which indicated that PTH may modulate the interoception signal in the spine. Collectively, our findings suggest that PTH treatment relieved pain through attenuation of nociceptive innervation in spinal degeneration.

### Osteoblasts are primarily responsible for PTH-mediated spine rejuvenation

To determine the primary cellular mediator of nociceptive innervation modification in response to PTH, we genetically deleted the PTH type 1 receptor (PPR) in osteoblasts and chondrocytes. PPR is widely expressed in the spine tissue, including chondrocytes, osteoblast lineage cells, and the intervertebral disc (IVD) (Supplemental Fig. 3A) and we have previously published that the pain relief effects were not mediated through the IVD ^[36]^. We first knocked out PPR in chondrocyte lineages using the inducible Col2a^ERT2^-Cre, resulting in PPR_Col2a_^ERT2−/−^ mice. Tamoxifen (100mg/kg) was intraperitoneally injected into PPR_Col2a_^ERT2−/−^ mice weekly, beginning at two months of age and continuing for one month. The efficiency of the genetic knockout was confirmed by immunofluorescence staining (Supplemental Fig. 3B). Mice then underwent LSI surgery and were injected with either PTH or vehicle daily for two months. Analysis via micro-CT of the L5 spine tissue unveiled that PTH continued to increase BV/TV and reduced both total porosity and pore space, relative to the vehicle-treated PPR_Col2a_^ERT2−/−^ LSI mice (Fig. 3, A–C). Similarly, both pressure tolerance test at lumbar region and Hargreaves test of the hind paw demonstrated a significant improvement in the PTH treated PPR_Col2a_^ERT2−/−^ LSI mice relative to the vehicle control (Fig. 3, D–E), though the active-wheel test showed no significant difference (Fig. 3F). In this case, we concluded that the chondrocyte lineage is not the primary target cell type responsible for PTH-mediated response in spinal degeneration. Knockout of PPR in the osteoblast lineage using Osteocalcin-Cre^[37]^, and LSI surgery (PPR_OC_^−/−^ LSI mice) ameliorated the effects of PTH relative to vehicle. Specifically, there was no significant difference in BV/TV, total porosity, or total pore space between PTH treatment and vehicle control in PPR_OC_^−/−^ LSI mice (Fig. 3G–I). PTH also no longer demonstrated efficacy in any of the three behavior tests (Fig. 3, J–L). Delving deeper into the innervation patterns, we found that no significant difference between the innervation of PGP9.5-positive and CGRP-positive nerve fibers in the vertebral bodies of PTH-treated PPR_OC_^−/−^ LSI mice relative to vehicle-treated PPR_OC_^−/−^ LSI mice (Fig. 3, M–O), nor did PTH treatment significantly change the expression of CGRP in DRG neuron of PPR_OC_^−/−^ LSI mice relative to vehicle (Fig. 3, P–Q). Therefore, our data strongly suggests that the osteoblast lineage cells are the principal cells responding to PTH treatment within our spine degeneration model.

### Nerve repelling factors secreted by Osteoblasts under PTH stimulation

**Figure 4:**
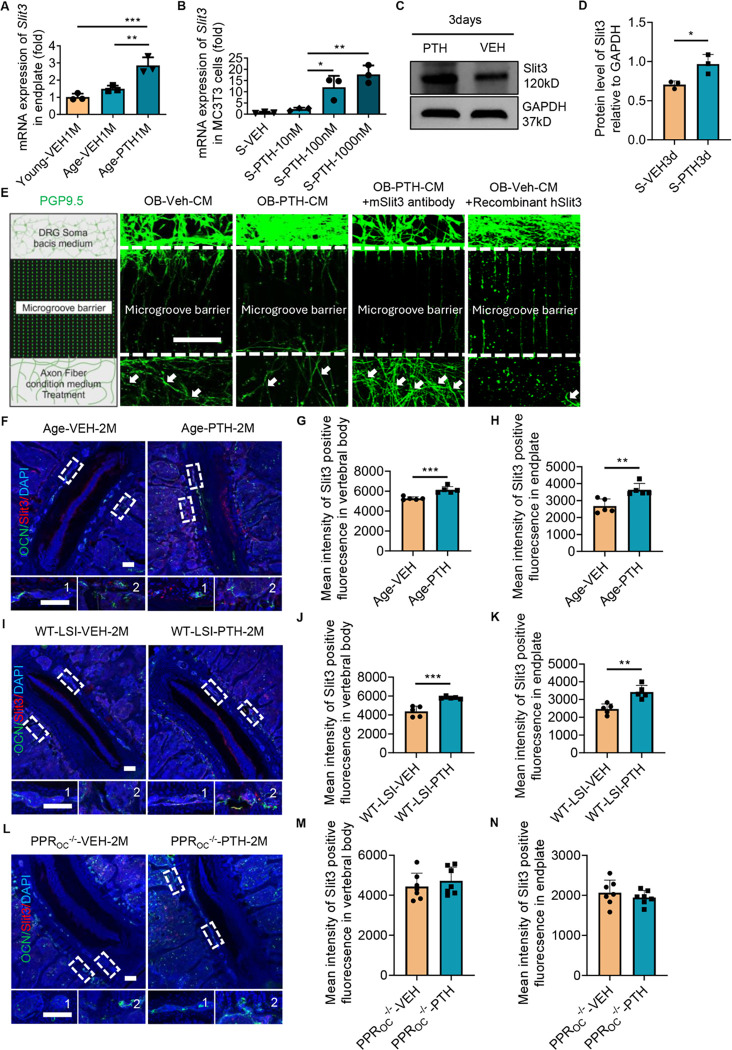
Osteoblasts Respond to PTH Treatment with increased Slit3 transcription and translation. **(A)** mRNA expression of the *Slit3* gene in the endplate tissue of WT young mice treated with vehicle (Veh) and aging mice treated with Veh or parathyroid hormone (PTH, 40μg/Kg/day) for one month. (n = 3, one-way ANOVA with Tukey’s multiple comparisons test). **(B)** mRNA expression of the *Slit3* gene in MC3T3 cells exposed to stimulated medium, and subsequently treated with Veh or PTH at different doses for 3 days. (n = 3, t-test). **(C-D)** Protein expression level of Slit3 in MC3T3 cells cultured with stimulated medium and treated with Veh or PTH for 3 days. (n = 3, t-test). **(E)** Representative image of immunofluorescence staining of PGP9.5 positive primary dorsal root ganglia (DRG) neuron fibers crossing the microgroove barrier (microfluid assay) cultured in condition medium (CM), CM+PTH, CM+PTH plus mouse Slit3 antibody (1 μg/ml), or CM+Veh (1X PBS) plus human recombinant Slit3 (1.25 μg/ml) for one week. **(F-N)** Representative images showing co-immunostaining for OCN (green) and Slit3 (red) in the lumbar spine sections and quantitative analysis of mean fluorescence intensity of Slit3 of aging mice (F-H), WT young mice two months after lumbar spine instability (LSI) surgery (I-K), and mice with conditional knockout of PPR in Osteocalcin (OC) expressing cells (PPR_OC_^−/−^) subjected to LSI surgery two months prior (L-N) and treated with PTH or Veh for two months. Scale bar: 100μm. (n ≥ 5, t-test). DAPI stains nuclei blue. 1: Endplate area; 2: Vertebral body area.

Nerve fiber growth is directed by various factors: Sema3a, EphrinB2, and Slit3 are known to function as nerve repelling factors^[38–40]^. To identify the potential repulsive guidance factor responsive to PTH treatment, we firstly extracted total mRNA from the spine endplate of young and aging WT mice treated with either PTH or vehicle. The qPCR results revealed the expression of repulsive factors genes *Slit3*, *Sema3a*, and *Efnb2* all significantly increased in PTH-treated aging mice relative to vehicle-treated aging mice; however, only *Slit3* in the PTH-treated aging mice was significantly higher relative to young mice (Fig. 4A; Supplemental Fig. 4, A-B). To explore the underlying mechanism of PTH-induced Slit3 secretion, we cultured MC3T3 cell line in osteoblast-inducing medium for three days. The qPCR analyses confirmed that the induced cells exhibited significantly elevated expression of *Bglap, Col1a1, Sp7*, and *Runx2* genes relative to unstimulated controls (Supplementary Fig. 4, C-F). Stimulated MC3T3 cells were then cultured in vehicle or PTH at various dosages in the presence of osteoblast-inducing medium for another three days; qPCR results indicated that PTH significantly increased *Slit3* expression in a dose-dependent manner (Fig. 4B). PTH inconsistently altered the gene transcription of *Sema3a* and *Efnb2*, increasing only at relatively lower dosages with suppression at the highest PTH concentration (Supplemental Fig. 4, G-H). Slit3 protein concentration also significantly increased in PTH (100nM) treated MC3T3 cells relative to vehicle control (Fig. 4, C–D).To further confirm the repulsive effect of PTH treatment MC3T3 cells, we cultured primary DRG neurons and conducted the microfluid assay using MC3T3 cell cultured condition medium with PTH or vehicle treatment, with or without Slit3 antibody treatment, as well as recombinant Slit3 treated negative control group. The results demonstrated that the length of primary DRG nerve axon significantly reduced in the PTH-condition medium-treated and Vehicle-condition medium-treated with recombinant hSlit3, relative to the Vehicle-condition medium-treated, while the PTH-condition medium-mSlit3 antibody treated group significantly increased nerve fiber growth relative to Vehicle-condition medium treated group (Fig. 4E). Increased expression of Slit3 was confirmed in vivo in both aging and WT LSI mice treated with PTH for two months relative to vehicle control (Fig. 4, F–K), whereas there was no difference in Slit3 between PTH and vehicle treated groups in PPR_OC_^−/−^ LSI mice (Fig. 4, L–N). Overall, we found that Slit3 served as the primary repulsive factor responding to PTH treatment.

### PTH stimulates Slit3 secretion from osteoblast through FoxA2

**Figure 5:**
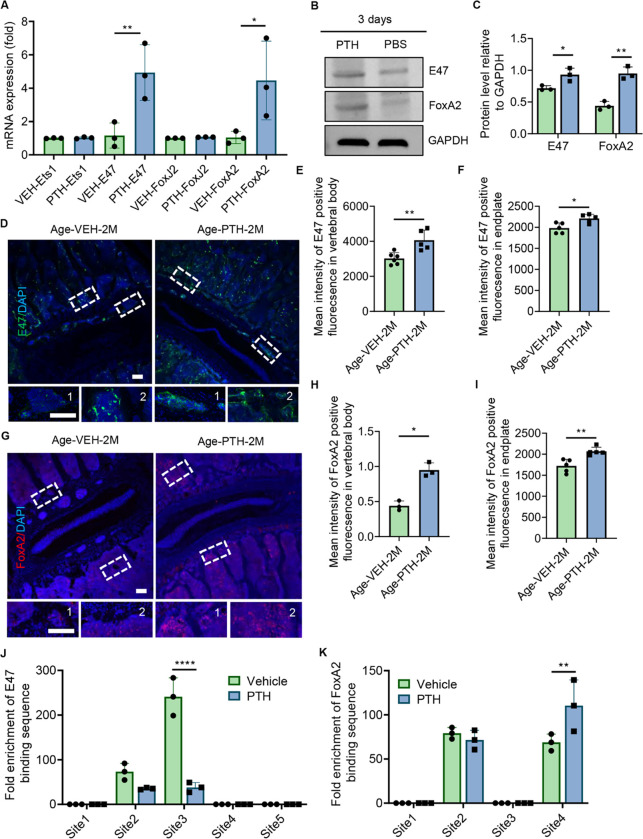
Transcriptional Mechanism Underpinning Slit3 Secretion. **(A)** mRNA expression levels of *Ets1, E47, FoxJ2*, and *FoxA2* genes in MC3T3 cells cultured in osteoblast differentiation-inducing medium and treated with vehicle or parathyroid hormone (PTH) 100 nM for 3 days. (n = 3, t-test). **(B-C)** Protein expression level of E47, FoxA2 in MC3T3 cells cultured in stimulated medium and treated with PTH 100 nM or phosphate buffered saline (PBS) for 3 days. (n = 3, t-test). **(D-F)**Representative images depicting E47 immunostaining (green) in lumbar vertebral body and endplate sections (D), quantitative analysis of the mean fluorescence intensity of E47 in the vertebral body and endplate (F) of aging mice, after a two-month treatment with Veh or PTH. Scale bar: 100μm. (n ≥ 5, t-test). 1: Endplate area; 2: Vertebral body area. **(G-I)** Representative images showing FoxA2 immunostaining (red) in lumbar vertebral body and endplate sections (G), quantitative analysis of the mean fluorescence intensity of FoxA2 in the vertebral body (H) and endplate (I) of aging mice treated with PTH or vehicle for two months. Scale bar: 100μm. (n ≥ 3, t-test). 1: Endplate area; 2: Vertebral body area. **(J)** Relative fold enrichment of the E47 binding site in the *Slit3* promoter region from stimulated MC3T3 cells treated with vehicle or PTH 100 nM for 3 days. (n = 3, two-way ANOVA with Sidak’s multiple comparisons test). **(K)** Relative fold enrichment of the FoxA2 binding site in the *Slit3* promoter region from stimulated MC3T3 cells treated with vehicle or PTH 100 nM for 3 days. (n = 3, two-way ANOVA with Sidak’s multiple comparisons test). DAPI stains nuclei blue.

Previous studies have indicated that the expression of *Slit3* can be regulated by transcription factors such as Ets1, E47, FoxJ2, and FoxA2^[41]^. To elucidate how PTH modulates Slit3 secretion in osteoblasts, we cultured MC3T3 cells in osteoblast differentiation-inducing medium and treated with PTH (100 nM) for another three days, followed by qPCR. Both *E47* and *FoxA2* mRNA were expressed at significantly higher levels in the PTH-treated group compared to the vehicle control (Fig. 5A). The protein concentration of E47 and FoxA2 also significantly increased in PTH treated cells relative to vehicle control in MC3T3 cells (Fig. 5, B–C). We then validated the expression of E47 and FoxA2 in the spine of aging mice. We observed that both E47 and FoxA2 were significantly upregulated in the spine endplate and vertebral body of aging mice treated with PTH for two months compared to those receiving vehicle treatment (Fig. 5, D–I). We performed the Chromatin Immunoprecipitation (ChIP) assay to confirm the transcriptional mechanisms regulating *Slit3* gene expression. While both E47 and FoxA2 regulated transcription through two distinct binding sites located on the *Slit3* gene promoter region, only one binding site of FoxA2 exhibited a significant increase in transcriptional binding affinity upon PTH stimulation (Fig. 5, J–K, Supplemental Fig. 5, A-B), suggesting that PTH treatment augments Slit3 secretion in osteoblast lineage cells primarily through FoxA2 transcriptional activation.

### Slit3 secreted by osteoblast contributes to spine rejuvenation with PTH treatment

**Figure 6:**
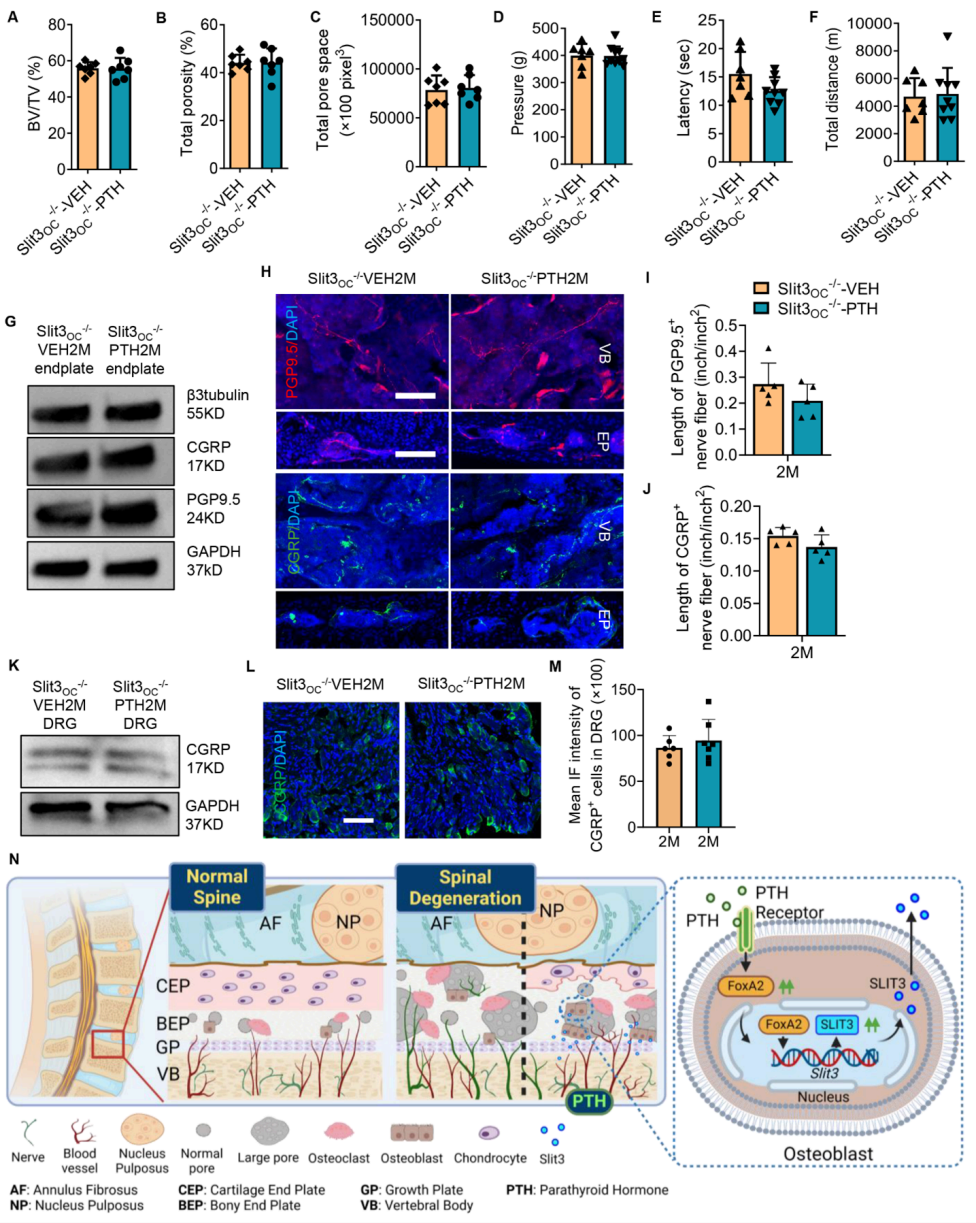
Efficacy of PTH Treatment is Diminished by Osteoblastic Slit3 Knockout in Mice with Spinal Degeneration. **(A-C)** Vertebral endplate bone structure analyses by micro-computed tomography: BV/TV percentage (A), Total porosity percentage (B), and Total pore space (C) in mice with conditional knockout of Slit3 in Osteocalcin (OC) expressing cells (Slit3_OC_^−/−^) subjected to lumbar spine instability (LSI) surgery two months prior to daily treatment with PTH (40μg/Kg/day) or vehicle (Veh) for two months. (n = 7, t-test). **(D-F)** Behavior evaluations included pressure tolerance of the lumbar spine assessed via the force threshold (D), latency of paw withdrawal post-thermal stimulation (E), and total distance covered in spontaneous activity in two days (F) for Slit3_OC_^−/−^ mice post LSI surgery for two months, with subsequent PTH or Veh for another two months. (n = 7, t-test). **(G)** Western blot analysis of protein expression levels of β3tubulin, CGRP, PGP9.5 relative to GAPDH in endplate tissue (L3-L5) in Slit3_OC_^−/−^ mice two months after LSI surgery and treated with PTH or Veh for an additional two months. (n = 5). **(H-J)** Representative images of PGP9.5 (red) and CGRP(green)-positive fibers (H), quantitative analysis of fiber length in the lumbar vertebral body of Slit3_OC_^−/−^ mice post-LSI and post-PTH or Veh treatment (I, J). DAPI stains nuclei blue. Scale bar: 100μm. (n ≥ 5, t-test). **(K)** Western blot analysis of protein expression levels of CGRP in dorsal root ganglia (DRG) tissue (L1-L2), normalized to GAPDH expression, in Slit3_OC_^−/−^ mice post LSI surgery for two months, and treated with PTH or Veh for an additional two months. (n = 5). **(L-M)** Representative immunofluorescence images showing CGRP-positive (green) neurons in DRG sections (L), followed by quantitative analysis of the mean immunofluorescence intensity for CGRP (M) in the DRG of Slit3_OC_^−/−^ mice post LSI surgery for two months and treated with PTH or Veh for another two months. Scale bar: 100μm. (n ≥ 6, t-test). **(N)** The schematic diagram demonstrates PTH increases transcriptional expression and secretion of Slit3 via FoxA2 in osteoblasts, repelling sensory nerves in the degenerated vertebral body and endplate, providing pain relief.

To confirm the significance of Slit3 secreted by osteoblast lineage cells in response to PTH treatment in spinal degeneration mice, we specifically knocked out the *Slit3* gene in osteoblast lineage cells, creating Slit3_OC_^−/−^ mice. Mice underwent LSI surgery at two months of age, two months later followed by treatment with either PTH or vehicle for another two months. Micro-CT analysis indicated no significant differences in vertebral endplate BV/TV, total porosity, or pore size between the PTH-treated mice and the vehicle-treated group (Fig. 6, A–C). Importantly, deletion of *Slit3* in osteoblasts negated the pain-relieving efficacy of PTH treatment, as evidenced by lack of significant difference between PTH and vehicle groups on behavior tests (Fig. 6, D–F). Furthermore, the protein extracted from the endplate (L5) of Slit3_OC_^−/−^ LSI mice revealed no differences in the expression levels of β3tubulin, PGP9.5 and CGRP in endplate tissues between PTH and vehicle treated groups (Fig. 6G). Similarly, there was no significant alteration of the peripheral sensory nerve fibers in the vertebral body or endplates between PTH and vehicle treated groups (Fig. 6, H–J). Neither the protein level nor the mean fluorescence intensity of CGRP in the L1/L2 DRG tissue demonstrated significant difference between PTH-treated Slit3_OC_^−/−^ LSI mice and the vehicle-treated control group (Fig. 6, K–M). Altogether, depletion of Slit3 in osteoblast lineage cells eliminated the efficacy of PTH treatment in spinal degeneration mice.

## Discussion

Low back pain (LBP) is a prevalent clinical problem with a series of complex etiologies based on the anatomy of spine, including spinal stenosis, facet arthropathy, myofascial pain, intervertebral disc degeneration, herniated nucleus pulposus, and endplate degeneration^[2, 42]^. We examined multiple mouse models with spinal hypersensitivity due to either spinal degeneration or instability and describe a unifying phenotype regarding LBP. We previously demonstrated that PTH treatment significantly improved spine degeneration and pain in the LSI surgery model and both aging models by reducing the local nociceptive innervation^[36]^. In the current study, we have further characterized the dynamic pathological characteristics of aging and LSI induced LBP in both bone structure and neuropathic activity. Most importantly, we demonstrate that PTH orchestrates nociceptive axon repulsion in the vertebral body and endplate by enhancing osteoblast *Slit3* transcription, repelling aberrant sensory innervation and alleviating pain. PTH treatment also resulted in a significantly decreased expression of CGRP and PGP9.5 within the DRG of both aged and LSI mouse models, underscoring the potential of PTH treatment in addressing the neuropathic components of low back pain in these conditions.

LBP may arise from disrupted equilibrium between osteoclast and osteoblast activities in the spinal vertebral region. Initially, a young, healthy endplate comprises chondrocytes embedded in a collagen matrix. Over time, these chondrocytes experience hypertrophy and ossification, leading to the formation of marrow-filled pores as a result of aging or degenerative processes^[43, 44]^. Both osteoclasts and osteoblasts are instrumental in pore formation and metabolic activities within this context. Bone homeostasis is regulated through the resorptive actions of osteoclasts and the formative functions of osteoblasts, mediated by cytokines such as TGF-β and IGF-1^[45, 46]^. Overactivity of osteoclasts can disrupt this balance, leading to uncoupling and pain in degenerative diseases like osteoarthritis and LBP.

We have previously shown that Netrin-1, secreted by osteoclasts, acts as a key nerve axon attractant factor, drawing nociceptive sensory innervation to the affected regions as observed in models of osteoarthritis and LBP^[11, 27]^. This study highlights that the nerve repulsive factor, Slit3, produced by the osteoblast lineage, counteracts the overactivity of osteoclasts facilitating sensory denervation, mitigating LBP.

The mechanism of nociceptive denervation is multifaceted and includes Slit3, Sema3a, and EphrinB2 as the major repulsive guidance molecules. Analysis of these factors revealed that only Slit3 exhibited a significant upregulation in PTH-treated aged mice relative to both vehicle-treated aged and younger mice. In contrast, the expression levels of Sema3a and EphrinB2 did not significantly differ between young and aged mice. Further supporting these findings, in vitro experiments revealed that high doses of PTH could suppress the expression of Sema3a and EphrinB2. We identified that the primary cell of PTH-stimulated Slit3 production is osteoblasts, rather than chondrocytes or cells within the IVD. We further clarified that the transcriptional mechanism of *Slit3* expression was regulated by FoxA2 and also related to E47. The binding affinity of E47 however was reduced in PTH-treated group even though it was still detectable, while the protein expression was even higher relative to the vehicle control in vivo. These results suggest that the transcriptional factor E47 may not stimulate *Slit3* transcription as the osteoblast response to PTH treatment. The increased expression of E47 in the spine section could instead from other cell types that respond to PTH treatment, and it may work for other pathways in PTH treated models.

The therapeutic efficacy of PTH in enhancing bone formation in osteopenic conditions such as osteoporosis is well-documented, and the underlying mechanisms have been extensively studied^[28, 30]^.Our findings suggest an additional mechanistic role in bone pain modification, particularly in degenerative spinal conditions as has been documented in animal models of osteoarthritis and LBP^[36, 47]^. This efficacy of analgesic effects of PTH has also been reported in human clinical trials, such as teriparatide and abaloparatide, synthetic analogs of human PTH and PTHr, respectively, where improvements in LBP were reported following treatment^[4, 48–50]^, although not always consistently^[51]^. The studies were not necessarily designed to assess changes in back pain, only recorded as an adverse event that occurred equally between teriparatide, abaloparatide, and placebo groups^[51]^. We also note that the inclusion criteria of these studies focused on osteopenia/osteoporosis and did not stratify by pathological changes of vertebral endplates which may be a critical criterion for future clinical trials.

Our study begins to elucidate potential mechanisms through which PTH alleviates pain. Our research posits that Slit3, acting as a critical nerve repulsive factor, plays a significant role in mitigating pain by repelling nerve fibers in the context of PTH treatment for LBP. Intriguingly, Slit3 has been identified as a factor promoting bone formation, secreted by osteoclasts^[52]^. Further research has positioned Slit3 as a proangiogenic factor derived from osteoblasts, essential for the CD31^hi^EMCN^hi^ endothelium, with its absence leading to reduced bone mass^[53]^. Both Slit3 and its receptor, Robo1, are implicated in bone metabolism and the maintenance of skeletal homeostasis^[54, 55]^. This dual role of Slit3, as elucidated in our study, suggests that PTH-induced elevation of osteoblast-derived Slit3 not only facilitates bone remodeling but also diminishes nociceptor innervation, thereby providing pain relief. Thus, Slit3 emerges as a promising therapeutic target for addressing bone degeneration issues, offering benefits from both skeletal and neuropathic perspectives.

This discovery elucidates the downstream mechanism of PTH treatment in LBP, demonstrating how it modulates the catabolic and anabolic balance between osteoclasts and osteoblasts to preserve bone homeostasis. Altogether, the pain signal in the degenerated spine region is transmitted by nociceptive nerve fibers, while the nociceptive innervation is regulated by the neuronal guidance factors, such as attractive factor Netrin-1 and repulsive factor Slit3, which are predominantly secreted by osteoclast and osteoblast, respectively. Abnormal bone coupling was triggered during the mechanical induced spine degeneration as well as aging, furthering aberrant innervation conducted by osteoclast activity. The excessive osteoclast function results in the secretion of Netrin-1, that could trigger the nociceptive pain by attracting nerve fiber growth. In our study, osteoblasts repel the nociceptive fibers and mitigate pain by secreting Slit3 in response to PTH treatment, while also reversing uncoupled bone remodeling (Fig. 6N). Therefore, the efficacy of PTH treatment in the spine degenerated pain is maintained by the coupling function of osteoblast and osteoclast in the vertebral region, and this mechanism could contribute to the clinic application of PTH for the LBP patients in the future.

## Method

### Animals models

The study utilized various mouse genotypes, including C57BL/6J (WT), SM/J, PPR_Col2a_^ERT2−/−^, PPR_OC_^−/−^, and Slit3_OC_^−/−^. We bought the WT young mice (#000664) and SM/J mice (#000687) from the Jackson Laboratory in USA, while obtained the WT aging mice (22 months of age) from National Institute on Aging in USA. The *Pth1r*(PPR) flox/flox mice were obtained from H. Kronenberg at Massachusetts General Hospital, located in Boston, MA, USA. We acquired the *Col2a*^ERT2^-Cre mouse line from the laboratory of Dr. Susan Mackem at Center for Cancer Research, NIH, Bethesda, Maryland, USA. The *Osteocalcin*(OC)-Cre mouse line was contributed by Thomas J. Clemens at Johns Hopkins University, located in Baltimore, Maryland, USA. We also acquired the *Slit3* flox/flox mouse line from Jung-Min Koh at University of Ulsan College of Medicine, located in Songpa-Gu, Korea. To accurately identify these genotypes, we performed polymerase chain reaction (PCR) analysis. This analysis involved extracting genomic DNA from the tails of the mice and utilizing a set of specific primers.

*Pth1r* Forward: 5′-TGGACGCAGACGATGTCTTTACCA-3′,

*Pth1r* Reverse: 5′-ACATGGCCATGCCTGGGTCTGAGA-3′;

*Col2a* ^ERT2^ Forward: 5′-GCGGTCTGGCAGTAAAAACTATC-3′,

*Col2a* ^ERT2^ Reverse: 5′-GTCAAACAGCATTGCTGTCACTT-3′;

*Osteocalcin* Transgene Forward: 5′-TCCTCAAAGATGCTCATTAG-3′,

*Osteocalcin* Transgene Reverse: 5′-GTAACTCACTCATGCAAAGT-3′,

*Osteocalcin* Internal positive control Forward: 5′-CAAATAGCCCTGGCAGAT-3′,

*Osteocalcin* Internal positive control Reverse: 5′-TGATACAAGGGACATCTTCC-3′;

*Slit3* Forward: 5′-GATTCTAAGAGCCTGCTTAG-3′,

*Slit3* Reverse: 5′-GACACTGGAGCGTAGGACTCC-3′.

In this study, Lumbar Spine Instability (LSI) surgery was conducted on adult male mice aged between two to three months. The mice groups included WT, PPR_Col2a_^ERT2−/−^, PPR_OC_^−/−^, and Slit3_OC_^−/−^ genotypes. The anesthesia protocol involved administering ketamine at a dosage of 100 mg/kg and xylazine at 10 mg/kg, the mixture was given intraperitoneally. The establishment of the LSI model in these mice was achieved through the surgical removal of the L3–L5 spinous processes, along with the supraspinous and interspinous ligaments, which was instrumental in creating LBP. In contrast, a sham procedure was performed on a different group of mice, which entailed only detaching the posterior paravertebral muscles from the L3–L5 vertebrae, without affecting the spine’s stability. Post-surgery, all mice were housed and cared for at the animal facility of The Johns Hopkins University School of Medicine. PTH (1–34, H-4835.0005, Bachem) treatment was intraperitoneally administered (40 μg/Kg/day) for two weeks, one month, or two months. The animal protocol was approved by the Institutional Animal Care and Use Committee of Johns Hopkins University, Baltimore, MD, USA

### Micro CT

The mice in the study were humanely euthanized through an overdose of isoflurane, followed by perfusion with 1X Phosphate-Buffered Saline (PBS) and 10% buffered formalin. For evaluating the endplates, we focused on the L5 segments of the lumbar spine; tissues were extracted and subjected to micro-Computed Tomography (μCT) analysis. The μCT parameters included a voltage of 55 kVp, a current of 181 μA, and a resolution of 9.0 μm per pixel, using a Skyscan 1172 system.

The μCT images were processed using the NRecon v1.6 software (Skyscan) for reconstruction. Quantitative assessments of these images were carried out using the CTAn v1.9 software (Skyscan). Regarding the endplates, we chose six consecutive images of the caudal endplates of L4-L5 and the L5 vertebrae in the coronal view. These images were utilized for 3D reconstruction using the CTVol v2.0 software (Skyscan).

### Pressure tolerance test

In our study, all behavioral assessments were conducted by an investigator who was not informed about the groupings of the mice. We utilized the SMALGO algometer (Bioseb) to measure pressure thresholds, which served as an indicator of pressure hyperalgesia. During the procedure, a sensor tip with a diameter of 5 mm was applied to the L4-L5 spinal region of each mouse. This was done while the mice were under gentle restraint. The pressure was incrementally increased at a rate of 50 grams per second until the mouse emitted a vocalization, indicating the threshold of pressure tolerance. This pressure force was recorded using the BIO-CIS software (Bioseb), with a maximum limit set at 500 grams to avoid causing any tissue damage. Between each testing session, the mice were given a 15-minute rest period to recover. The average of these measurements was then calculated to determine the final pressure tolerance threshold for each mouse.

### Active wheel test

For the assessment of spontaneous wheel-running activity, we employed specialized mouse activity wheels (BIO-ACTIVW-M model, Bioseb). This setup included software capable of accurately tracking and recording each mouse’s activity levels within the wheel cage. Prior to the commencement of testing, mice were allowed an overnight period to acclimatize to the wheel cage environment. During the testing period, the mice experienced a 12-hour light/dark cycle. Each mouse was monitored in this setup for a continuous period of 48 hours. Throughout this duration, the software automatically logged various parameters pertaining to their spontaneous activity levels.

### Hargreaves test

In our study, the Hargreaves method was employed to evaluate analgesia levels in various groups of mice. Each group of mice was first given an hour to become accustomed to the testing environment. For the test, a focused beam of radiant heat (provided by IITC Life Science Inc.) was directed onto the plantar surface of the hind paws of the mice. The response time, assessed as the time duration until the mouse withdrew its paw, was carefully measured. This response time, indicative of the latency period to the heat stimulus, was recorded for each paw. To ensure accuracy and consistency, this procedure was repeated a minimum of five times per mouse. The average of these latency times was then calculated and used for subsequent analysis.

### Immunofluorescence staining

Upon euthanasia, bone specimens, specifically the L3-L5 lumbar spine, were harvested and immediately fixed in 10% buffered formalin for a duration of 24 hours. The L1-L2 DRG tissues were isolated and fixed in 10% buffered formalin overnight. Subsequently, the bone samples underwent a decalcification process at a temperature of 4°C. This was achieved using 0.5M ethylenediaminetetraacetic acid (EDTA) for a period of three weeks, accompanied by constant agitation. The samples were embedded in O.C.T. Compound embedding medium (Sakura).

For histological examination, we prepared 40 μm thick sections of spine tissue or 10 μm thick of DRG tissue sections for immunofluorescence staining following our previous protocol^[56]^. The spine sections were incubated for 48 hours at 4°C with primary antibodies targeting CGRP (1:100, ab81887, Abcam), PGP9.5 (1:200, SAB4503057, Sigma), incubated overnight at 4°C with primary antibodies targeting Osteocalcin (1:200, M188, Takara), PTH1R (1:100, ab75150, Abcam), Slit3 (1:100, AF3629, Biotechne), FoxA2 (1:100, 22474–1-AP, Proteintech), and E47 (1:100, sc-416, Santa Cruz), while the DRG sections were incubated overnight at 4°C with primary antibodies targeting CGRP (1:100, ab81887, Abcam) and β3tubulin (1:100, 2G10, Thermo Fisher). These were followed by the application of appropriate secondary antibodies and DAPI (1:250, H-1200, Vector) for one hour in a light-protected environment. For the visualization and documentation of the samples, we employed both a fluorescence microscope (Olympus BX51, DP71) and a confocal microscope (Zeiss LSM 880). Quantitative analyses of the images were performed using ImageJ software (National Institutes of Health, Bethesda, MD).

### Western blot

We pulverized the endplate tissue samples in a liquid nitrogen environment to facilitate the extraction of total protein. This extraction was carried out using the T-PER^™^ Tissue Protein Extraction Reagent (catalog number 78510, Thermo Fisher), complemented with 1% Protease and Phosphatase Inhibitor cocktail (catalog number 78442, Thermo Fisher). For cell culture lysates, we utilized RIPA buffer (catalog number 89901, Thermo Fisher), also supplemented with 1% of the aforementioned Cocktail. The lysates obtained were then centrifuged and their protein concentrations standardized using the BCA Protein Assay Kit (catalog number 23227, Thermo Fisher).

The protein samples prepared were subsequently resolved by electrophoresis on a 10% SDS-PAGE gel and transferred to polyvinylidene difluoride membranes (sourced from Bio-Rad Laboratories). The membranes, post-transfer, were blocked with 5% fat-free milk and incubated overnight with specific primary antibodies at 4°C. Following this, the membranes were washed with Tris-buffered saline mixed with 0.05% Tween-20 (TBST) and incubated with horseradish peroxidase (HRP)-conjugated secondary antibodies.

For protein detection, we employed an enhanced chemiluminescence kit provided by Thermo Fisher Scientific. A range of primary antibodies was used for this purpose, including those specific for mouse β3tubulin (1:500, 2G10, Thermo Fisher), CGRP (1:1000, sc-57053, Santa Cruz), PGP9.5 (1:1000, SAB4503057, Sigma), IB4 (1:1000, I21441, Thermo Fisher), TH (1:1000, AB152, Sigma), Slit3 (1:1000, AF3629, Biotechne), E47 (1:1000, sc-416, Santa Cruz), FoxA2 (1:1000, 22474–1-AP, Proteintech), and GAPDH (1:2000, 14C10, Cell Signaling), which facilitated the determination of protein concentrations in the lysates.

### MC3T3 cell culture

MC3T3 subclone 4 cell line was purchased from ATCC (CRL-2593^™^) and cultured using Alpha Minimum Essential Medium with ribonucleosides, deoxyribonucleosides, 2 mM L-glutamine and 1 mM sodium pyruvate, but without ascorbic acid (A10490–01, Thermo Fisher). The osteoblast differentiation medium was supplied with 50 μg/ml ascorbic acid (Sigma) and 2 mM of β-glycerophosphate (G9422, Sigma) to induce osteoblast differentiation for three days. The PTH (1–34) was diluted in 1X PBS into different does for cell treatment. Cells were cultured with 10% fetal bovine serum (35–011-CV, Sigma-Aldrich) at 37°C in a 5% CO_2_-humidified incubator.

### qPCR test

The total RNA was extracted from the spine endplate tissue or cultured cells using RNeasy Plus Mini Kit (74134, Qiagen) according to the manufacturer’s instructions. The purity of RNA was tested by measuring the ratio of absorbance at 260 nm over 280 nm. For RT-PCR, 500ng of RNA was reverse transcribed into complementary DNA using the PrimeScript^™^ RT Master Mix (RR036A, Takara), then RT-PCR was performed with SYBR Green-Master Mix (Qiagen) using QuantStudio 3 Real-Time PCR System (Thermo Fisher). Relative expression was calculated for each gene by the 2^−ΔΔ^ CT method, with glyceraldehyde 3-phosphate dehydrogenase (*Gapdh*) for normalization as we reported^[57]^. Primers used for RT-PCR are listed as below:

*Slit3* Forward: 5′-TGCCCCACCAAGTGTACCT-3′,

*Slit3* Reverse: 5′-CGCCTCTCTCGATGATGCT-3′;

*Sema3a* Forward: 5′-CACTGGGATTGCCTGTCTTTT-3′,

*Sema3a* Reverse: 5′-TGGCACATTGTTCTTTCCGTTT-3′;

*Efnb2* Forward: 5′-GCTAGAAGCTGGTACAAATGGG-3′,

*Efnb2* Reverse: 5′-CATCGGTGCTAGAACCTGGA-3′;

*Bglap* Forward: 5′-CTGACCTCACAGATCCCAAGC-3′,

*Bglap* Reverse: 5′-TGGTCTGATAGCTCGTCACAAG-3′;

*Col1a1* Forward: 5′-GCTCCTCTTAGGGGCCACT-3′,

*Col1a1* Reverse: 5′-CCACGTCTCACCATTGGGG-3′;

*Sp7* Forward: 5′-ATGGCGTCCTCTCTGCTTG-3′,

*Sp7* Reverse: 5′-TGAAAGGTCAGCGTATGGCTT-3′;

*Runx2* Forward: 5′-ATGCTTCATTCGCCTCACAAA-3′,

*Runx2* Reverse: 5′-GCACTCACTGACTCGGTTGG-3′;

*Ets1* Forward: 5′-TCCTATCAGCTCGGAAGAACTC-3′,

*Ets1* Reverse: 5′-TCTTGCTTGATGGCAAAGTAGTC-3′;

*E47* Forward: 5′-GGGTGCCAGCGAGATCAAG-3′,

*E47* Reverse: 5′-ATGAGCAGTTTGGTCTGCGG-3′;

*FoxJ2* Forward: 5′-GCCTCCGACCTGGAGAGTAG-3′,

*FoxJ2* Reverse: 5′-CTGTACCGTGGCTTGCCAT-3′;

*FoxA2* Forward: 5′-CCCTACGCCAACATGAACTCG-3′,

*FoxA2* Reverse: 5′-GTTCTGCCGGTAGAAAGGGA-3′;

*Gapdh* Forward: 5′-CATCACTGCCACCCAGAAGACTG-3′,

*Gapdh* Reverse: 5′-ATGCCAGTGAGCTTCCCGTTCAG-3′.

### Primary DRG neuron isolation and culture

The young WT mice were euthanized as described above for the DRG tissue isolation. We dissected the DRG tissue from thoracis and lumbar vertebra under microscope and collected in F12 Minimum Essential Medium (F12-MEM, Gibco) supplemented with 1% Penicillin-Streptomycin solution (P.S.) at 4 °C. The medium was then replaced by 1 ml collagenase Type I solution (1 mg/ml, 17100017, Gibco) and incubated in a microfuge tube at 37°C for 90 min. Collagenase solution was then replaced with 500 μl 1X TrypLE^™^ Express Enzyme solution (12604013, Gibco) and incubated at 37°C for 15 min. Specimen was centrifuged and the tissue pellet was collected (1000 rpm, 5 mins). The pellet was resuspended using F12-MEM medium containing 1X supplement B27 (17504044, Gibco) and filtered using 40 μm strainer. Prior to use in experiments, the DRG neurons were collected by centrifuge under 1000 rpm for 5 mins.

### Microfluid assay

For our neuron culture studies, we employed the Innsbruck Neuron Device (IND500) featuring a 500-μm microgroove barrier. This device was set up on a Corning cell culture dish with a 10 cm diameter. Initially, the device underwent a cleaning process involving an overnight soak in 10% hydrochloric acid, followed by a thorough ultrasonic cleaning in distilled and deionized water, repeated three times for 20 minutes each session. Prior to each experimental run, the device was air-dried and placed onto the culture dish. The dish wells were prepared by applying 100 μl of a coating solution that contained 100 μg/ml Poly-D-Lysine for one hour at 37°C, then coated with 10 μg/ml Laminin to each well after 1X PBS washing five times. The plate was incubated at 37°C for one hour, then the coating solution was discarded, and the wells were rinsed thrice with sterile 1X PBS.

DRG neurons were introduced into the central channel of the device. The successful migration of neurons into the designated channel was confirmed via microscopy. Subsequently, about 150 μl of culture medium was dispensed into each side well and cultured for three days before further intervention. Then different interventions were administered to the wells: 150 μl conditioned medium from vehicle or PTH-treated osteoblasts, with or without Slit3 antibody (1 μg/ml, AF3629, R&D Systems), or human recombinant Slit3 protein (1.25 μg/ml, 9067-SL, Biotechne), for one week. Nerve growth factor (50 ng/ml, N-100, Alomone Labs) was supplemented for each well. After one week of incubation, the neurons and their axons were fixed and prepared for immunofluorescence staining.

For staining, the culture medium was removed, and cells were fixed using 4% paraformaldehyde (PFA, 200 μl/well) for 15 minutes at room temperature. Following fixation, the cells underwent three 1X PBS washes and were blocked with a solution containing 1% bovine serum albumin, 0.3% Triton X-100, and 2% normal goat serum in 1X PBS for an hour at room temperature. Axons were labeled with PGP9.5 antibody (1:200, SAB4503057, Sigma) and incubated overnight at 4°C. Post-secondary antibody treatment, the wells were washed and prepared for confocal microscopy analysis using a Zeiss LSM 880 system.

### Chip assay

MC3T3 cells cultured with osteoblast differentiation medium for three days and treated with PTH (100nM) or vehicle for another three days. Chip assay was performed according to the manufacturer’s protocol (Pierce^™^ Agarose CHIP Kit, Cat. 26156, Thermo Fisher). Briefly, we crosslinked the cell pellet using Glycine Solution after fixation in 1% formaldehyde. The cells were lysed in membrane extraction lysis buffer and nuclear extraction lysis buffer, along with MNase digestion (DTT, MNase Digestion Buffer). Of the sample, 10% was removed as an input control. Antibodies targeting E47 (sc-416, Santa Cruz), FoxA2 (22474–1-AP, Proteintech) were utilized. Additionally, anti-RNA polymerase II and control IgG served as the positive and negative controls, respectively. The DNA samples were further analyzed by qPCR and electrophoresis as introduced by the manufacture. The PCR primers used to detect E47 and FoxA2 binding site were as follows:

E47 Site #1: Forward: 5′-TCAGCCCTGGTACTAAAT-3′,

Reverse: 5′-CAAACCTTGAACCAATTT-3′;

E47 Site #2, Forward: 5′-GAGGACTGAGGCAAAGGC-3′,

Reverse: 5′-CTCTGCTTCCGATGGTGA-3′;

E47 Site #3, Forward: 5′-AGGCTATTTCAGACCTTT-3′,

Reverse: 5′-CAGGCTCCATACATACTTG-3′.

E47 Site #4, Forward: 5′-AGAACGGTGGCACCTTGA-3′,

Reverse: 5′-GCGGACCTTTATTTCCTTATTT-3′.

E47 Site #5, Forward: 5′-CCTACAGGCTCTTGGTTGCTC-3′,

Reverse: 5′-CGCTCGCTTTCTCCATTCAC-3′.

FoxA2 Site #1: Forward: 5′-TGGGGGTGGGGGGGGGGAGCTGGGG-3′,

Reverse: 5′-TCTTCTATTTTCCTTAAAGGAAACT-3′;

FoxA2 Site #2, Forward: 5′-TCAAGGAAGTCTGGGCAATA-3′,

Reverse: 5′-GGCAGGAACTGGAGGAAA-3′;

FoxA2 Site #3, Forward: 5′-TAGTTGTTGGCCTTAGCT-3′,

Reverse: 5′-TGAAATGATTATCCGAGAC-3′.

FoxA2 Site #4, Forward: 5′-GGGAGGCGGAGCTGGTGTTT-3′,

Reverse: 5′-GCGCTCGCTTTCTCCATTCAC-3′.

### Statistics

Statistical evaluations were conducted utilizing GraphPad Prism version 8.0 (Boston, MA, USA), with outcomes expressed as the mean ± standard deviation. Differences among multiple experimental groups were assessed using one-way Analysis of Variance (ANOVA) followed by Tukey’s multiple comparison test. Comparisons between two distinct groups were using an unpaired, two-tailed Student’s t-test. A *P*-value of less than 0.05 was designated as the threshold for statistical significance across all experimental conditions.
